# Comparison of 24 Versus 46-Hour 5-FU Infusion Durations in the Perioperative FLOT Therapy for Gastric Cancer

**DOI:** 10.3390/medicina62081444

**Published:** 2026-07-25

**Authors:** Savas Gokcek, Pervin Can Sancı, Anıl Karakayalı, Emine Bihter Eniseler, Melis Kılınc, Ozge Yetginoglu, Ali Kalem, Caglar Koseoglu, Ferhat Ekinci, Mehmet Uzun, Huseyin Salih Semiz, Ilkay Tugba Unek

**Affiliations:** 1 Department of Medical Oncology, Kahramanmaraş Necip Fazıl City Hospital, 46040 Kahramanmaraş, Türkiye; 2 Department of Medical Oncology, Faculty of Medicine, Kocaeli University, 41380 Kocaeli, Türkiye; ppervincan@hotmail.com (P.C.S.); anilozbay@hotmail.com (A.K.); 3 Department of Medical Oncology, Faculty of Medicine, Celal Bayar University, 45030 Manisa, Türkiye; drbihtereniseler@gmail.com (E.B.E.); drferhatekinci@hotmail.com (F.E.); 4 Department of Medical Oncology, Faculty of Medicine, Dokuz Eylül University, 35340 İzmir, Türkiye; drmeliskilinc@gmail.com (M.K.); ozyetginoglu@gmail.com (O.Y.); hsalihsemiz@hotmail.com (H.S.S.); ilkaytugbaunek@gmail.com (I.T.U.); 5 Department of Medical Oncology, Faculty of Medicine, Gaziantep University, 27310 Gaziantep, Türkiye; kalemali88@gmail.com; 6 Department of Medical Oncology, Gaziantep City Hospital, 27310 Gaziantep, Türkiye; drcaglarkoseoglu@hotmail.com; 7 Department of Medical Oncology, Tepecik Training and Research Hospital, University of Health Sciences, 35340 İzmir, Türkiye; memed.uzun3846@gmail.com

**Keywords:** gastric cancer, FLOT regimen, 5-fluorouracil, pathological response, perioperative chemotherapy

## Abstract

*Background and Objectives*: This study aimed to evaluate the effects of 24 h versus 46 h continuous 5-fluorouracil (5-FU) infusion durations on pathological response, progression-free survival (PFS), and overall survival (OS) in patients with gastric and gastroesophageal junction adenocarcinoma receiving a perioperative FLOT (fluorouracil, leucovorin, oxaliplatin, and docetaxel) regimen. *Materials and Methods*: A total of 300 patients who received a perioperative FLOT regimen were retrospectively analyzed. The patients were divided into two groups as those receiving 24 h and 46 h continuous 5-FU infusion. Clinicopathological characteristics were compared. Survival analyses were evaluated using the Kaplan–Meier method. Factors affecting pathological response were analyzed by logistic regression, and factors affecting survival were examined by Cox regression analysis. *Results*: Pathological response rates were found to be significantly higher in patients receiving 46 h 5-FU infusion (multivariate analysis: OR = 2.884; 95% CI: 1.619–5.138; *p* < 0.001). The probability of pathological response was found to be significantly lower in patients with baseline cT4 and advanced nodal stage. Median OS times were 41.23 months and 33.73 months in the 24 h and 46 h groups, respectively, with no significant difference detected between the groups (*p* = 0.371). In contrast, median PFS time was significantly longer in the 24 h group (38.47 months vs. 18.00 months; *p* < 0.001). However, in multivariate analysis, 5-FU infusion duration could not be demonstrated as an independent prognostic factor for PFS. Moderately and poorly differentiated tumors and an increased number of positive lymph nodes were found to be independent risk factors for progression. *Conclusions*: Although 46 h 5-FU infusion in the perioperative FLOT regimen was associated with a higher pathological response rate, this advantage was not reflected in survival outcomes. It is considered that the observed difference in PFS may stem from the clinicopathological heterogeneity between the groups. Prospective randomized studies are needed to determine the optimal 5-FU infusion duration.

## 1. Introduction

Gastric cancer (GC) remains one of the leading causes of cancer-related mortality worldwide, despite advances in diagnosis and treatment [1]. Although surgical resection performed with appropriate lymph node dissection forms the cornerstone of curative treatment in localized disease, recurrence rates remain high, particularly in locally advanced disease [2]. Therefore, perioperative systemic treatment approaches have become an essential component of modern GC management. The treatment landscape for gastric adenocarcinoma requires a comprehensive multidisciplinary approach, beginning with precise clinical staging and diagnostic workup. High-quality imaging, endoscopy, and biopsies are critical not only to determine localized versus diffuse disease but also to establish tumor grade and microbiological characteristics. Based on these diagnostic findings, the multidisciplinary team evaluates treatment strategies, taking into crucial account the patient’s nutritional support, eligibility for neoadjuvant chemotherapy, or the indication for upfront surgery. For patients with advanced disease, diagnostic laparoscopy serves as a prerequisite to rule out occult peritoneal carcinomatosis, potentially selecting candidates for pressurized intraperitoneal aerosol chemotherapy. Currently, the standard of care for resectable, locally advanced gastric cancer relies heavily on neoadjuvant/perioperative regimens like the triplet FLOT or docetaxel-based combinations (DOCs) [3]. Furthermore, modern treatment decisions are increasingly shaped by tumor biology, incorporating targeted therapies and immunotherapeutic agents based on human epidermal growth factor receptor 2 (HER2) status, microsatellite instability (MSI), and newer biomarkers such as claudin 18.2.

It has been demonstrated by various studies that perioperative chemotherapy provides a survival advantage in resectable gastric and gastroesophageal junction (GEJ) adenocarcinomas. Early randomized trials, such as the MAGIC trial, revealed that perioperative ECF (Epirubicin, Cisplatin, 5-FU)-based treatments improved survival compared to surgery alone [4]. However, due to limited pathological response rates and treatment-related toxicity, the search for more effective regimens has continued. Beyond clinical staging, emerging translational research highlights that prognostic heterogeneity in gastric cancer is heavily driven by molecular features, lineage plasticity, and epithelial–mesenchymal transition (EMT) pathways. For instance, cell adhesion and differentiation-associated proteins, such as V-set and immunoglobulin domain-containing 1 (VSIG1), have recently been identified as crucial regulators of the epithelial phenotype in gastric tumors, where their loss or downregulation serves as an emerging marker for EMT and tumor dedifferentiation [5]. Undergoing EMT allows tumor cells to acquire a highly invasive, chemoresistant, and poorly cohesive phenotype, which fundamentally shapes the biological behavior and treatment response of aggressive clinical subtypes.

In this process, the FLOT regimen, consisting of a combination of fluorouracil, leucovorin, oxaliplatin, and docetaxel, has become the standard perioperative treatment approach in locally advanced resectable GC and GEJ adenocarcinomas following the results of the FLOT4-AIO trial [6]. The FLOT regimen has been shown to provide higher pathological response rates and longer overall survival compared to older anthracycline-based regimens [6,7].

However, some practical details regarding the administration of the FLOT regimen continue to vary among centers. One of these variations is the infusion duration of 5-fluorouracil (5-FU), which is administered as a continuous infusion. 5-FU is a time-dependent antimetabolite, and it is known that the antitumor efficacy of the drug can vary depending on exposure duration and administration schedule [8]. It has been reported that continuous infusion administrations may produce different pharmacodynamic characteristics and toxicity profiles compared to bolus administrations [9].

In clinical practice, during perioperative FLOT treatment, some centers administer a 24 h continuous 5-FU infusion, while others apply a 46 h continuous 5-FU infusion. These specific infusion durations—24 h and 46 h—are established standards derived from validated oncology protocols. The 24 h schedule represents the original FLOT4 trial design, whereas the 46 h schedule is a widely accepted clinical modification adapted from the standard infusional 5-FU dosing schedules of the FOLFOX and FOLFIRINOX regimens. Other non-standard durations (such as 36 or 48 h) are not utilized in clinical practice as they lack clinical trial validation and do not align with standardized ambulatory pump programming. However, data comparing the effects of these two different administration durations on pathological response, progression-free survival (PFS), and overall survival (OS) are highly limited. In particular, the impact of 5-FU infusion duration on neoadjuvant treatment response has not been clearly established.

In this study, we aimed to compare the effects of 24 h versus 46 h continuous 5-FU infusion administrations on pathological response, PFS, and OS in patients with gastric cancer receiving perioperative FLOT treatment. Additionally, we aimed to evaluate the clinicopathological prognostic factors influencing pathological response and survival outcomes.

## 2. Materials and Methods

### 2.1. Study Design and Patient Selection

A total of 300 patients diagnosed with locally advanced resectable gastric and gastroesophageal junction adenocarcinoma who received the perioperative FLOT regimen were pooled from 5 oncology centers for this study. The study population consisted of patients who underwent curative surgical resection after neoadjuvant FLOT treatment and whose postoperative pathological evaluations were completed. Patients were divided into two groups based on the continuous 5-FU infusion duration administered: (a) patients receiving 24 h continuous 5-FU infusion, and (b) patients receiving 46 h continuous 5-FU infusion. As this was a retrospective study, patients were not prospectively selected or assigned to a specific infusion cohort by the investigators. The selection of either the 24 h or 46 h 5-FU infusion schedule was entirely determined by individual institutional preferences, local nursing workflows, and the clinical discretion of the treating oncologists at each participating center. Inclusion criteria were defined as: histopathologically confirmed presence of gastric or gastroesophageal junction adenocarcinoma, administration of perioperative FLOT regimen, history of curative surgical resection, and complete access to clinicopathological and survival data. Patients with missing data, those receiving palliative treatment due to metastatic disease, or those who did not undergo surgery were excluded from the study.

### 2.2. Data Collection

Patients’ demographic, clinical, pathological, and treatment-related data were retrospectively obtained from the hospital electronic medical record system and patient files. Variables evaluated in the study included age, sex, body mass index (BMI), baseline clinical tumor (T) and node (N) stage, tumor localization, histopathological subtype, Lauren classification, tumor differentiation grade, type of surgery, type of lymph node dissection, number of harvested lymph nodes, pathological stage, HER2 status, immunohistochemical status, and MSI status.

All patients were evaluated according to the American Joint Committee on Cancer (AJCC) TNM (Tumor–Node-Metastasis) staging system. Pathological evaluations were performed by experienced gastrointestinal pathologists. Additionally, where available in the medical records, treatment delivery parameters—including the completion rates of planned perioperative FLOT cycles, treatment delays (defined as a delay of more than 7 days), dose reductions (any dose reduction of docetaxel, oxaliplatin, or 5-FU), and clinically significant grade 3–4 toxicities—were collected and analyzed.

### 2.3. Study Endpoints

The primary endpoint of the study was defined as the comparison of pathological response rates between the 24 h and 46 h continuous 5-FU infusion groups. Secondary endpoints were defined as: OS, PFS, prognostic factors affecting pathological response, and evaluation of prognostic factors influencing OS and PFS.

OS was defined as the time from the date of diagnosis to the date of death due to any cause. PFS was calculated as the time from the initiation of treatment to the development of radiological progression or death.

### 2.4. Statistical Analysis

Data analysis was performed using IBM SPSS Statistics for Windows, Version 26.0 (IBM Corp., Armonk, NY, USA), and a 95% confidence interval was accepted in all analyses. The conformity of continuous variables to normal distribution was evaluated using skewness and kurtosis values. Skewness and kurtosis values between −3 and +3 were considered sufficient for normal distribution. The independent samples t-test was used for comparing normally distributed continuous variables between the two groups. For the analysis of non-normally distributed continuous variables, the Mann–Whitney U test was preferred. The Chi-square test was used to compare categorical variables.

Logistic regression analysis was used to determine the factors affecting pathological response. Survival analyses were performed using the Kaplan–Meier method, and the groups were compared with the log-rank test. Cox regression analysis was used to evaluate prognostic factors influencing OS and PFS. A *p*-value of <0.05 was considered statistically significant. For the multivariable logistic regression and Cox proportional hazard regression models, candidate variables were selected based on a univariable significance threshold of p<0.10. Additionally, key established clinical prognostic factors, including pathological tumor stage (T and N stage), histological differentiation, and resection margin status (R0 vs. R1), were entered into the multivariable models as forced covariates regardless of their univariable *p*-values to control for potential clinical confounding.

## 3. Results

A total of 300 patients were included in the study. While 150 (50%) of the patients received 24 h continuous 5-FU infusion, 150 (50%) received 46 h continuous 5-FU infusion. The clinicopathological characteristics of the patients are summarized in Table 1.

The mean age of the patients was 63.72 ± 10.1 years, and the mean age of patients receiving 46 h 5-FU was found to be significantly lower than that of patients receiving 24 h 5-FU (59.17 ± 10.12 vs. 63.72 ± 10.1; *p* < 0.001) (Table 1). No significant difference was observed between the groups in terms of body mass index (*p* = 0.462).

When the number of positive lymph nodes was evaluated, the median number of positive lymph nodes was found to be higher in the 46 h 5-FU group [3 (Q25–Q75: 0–7) vs. 1 (Q25–Q75: 0–4); *p* < 0.001].

A significant difference was found between the groups in terms of stage distribution at the time of diagnosis (*p* = 0.046). The proportion of stage III patients was found to be higher in the 46 h infusion group (71.3% vs. 61.3%). Similarly, a significant difference was observed in terms of tumor localization (*p* = 0.028). GEJ tumors were detected at a higher rate in the 46 h group (19.4% vs. 7.3%).

When the histopathological features were examined, the rate of signet ring cell carcinoma was found to be higher in the 46 h group (22% vs. 13.3%; *p* = 0.007). According to the Lauren classification, intestinal-type tumors were more common in the 24 h group (69.3% vs. 32.3%), while the unknown Lauren subtype was higher in the 46 h group (*p* < 0.001).

When evaluated in terms of surgical characteristics, the rate of total gastrectomy was significantly higher in the 46 h group (76.6% vs. 52%; *p* < 0.001). Similarly, the rates of D2 lymph node dissection (86.3% vs. 42.7%; *p* < 0.001) and ≥16 harvested lymph nodes (80.7% vs. 38%; *p* < 0.001) were higher in the 46 h group (Table 1).

When the pathological nodal status was examined, the ypN0 rate was higher in the 24 h group (45.3% vs. 26.7%), while the rate of ≥7 lymph node metastases was found to be higher in the 46 h group (28.7% vs. 11.3%; *p* < 0.001).

**Figure 1:**
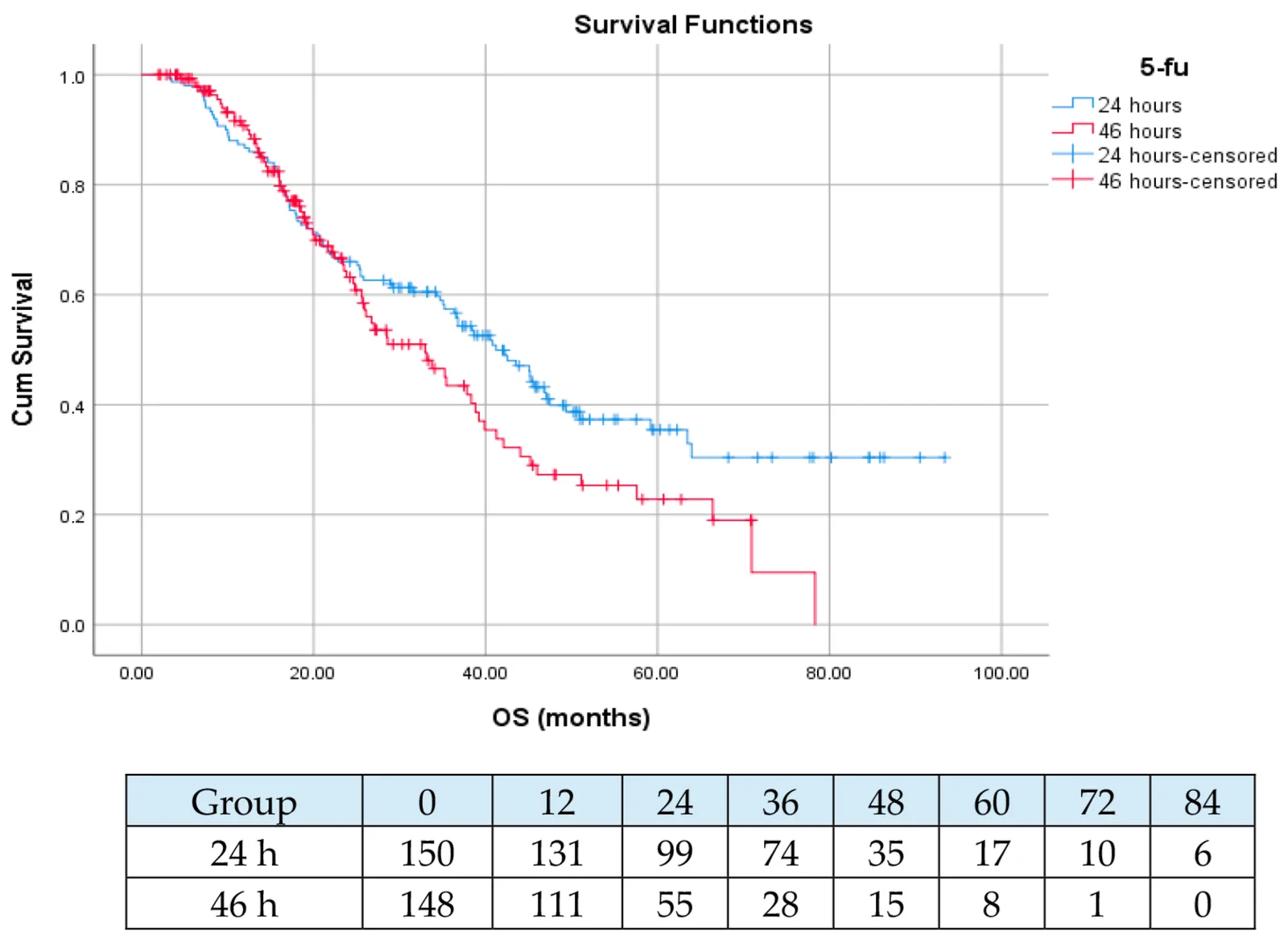
Kaplan–Meier survival curves for overall survival (OS) comparing the 24 h and 46 h 5-FU infusion groups. The table beneath the curves displays the number of patients at risk at each specified time point.

According to the analysis results, the median overall survival (OS) for patients receiving the 24 h 5-FU infusion was 41.23 months (95% CI: 35.10–47.36), whereas it was 32.97 months (95% CI: 24.48–41.46) for patients receiving the 46 h 5-FU infusion. No statistically significant difference in overall survival was detected between the two groups (*p* = 0.058). This finding indicates that the duration of the 5-FU infusion (24 h versus 46 h) does not have a statistically significant impact on overall survival. The number of patients at risk at 0, 12, 24, 36, and 48 months was 150, 131, 99, 74, and 35 in the 24 h group, and 148, 111, 55, 28, and 15 in the 46 h group, respectively (Figure 1).

**Figure 2:**
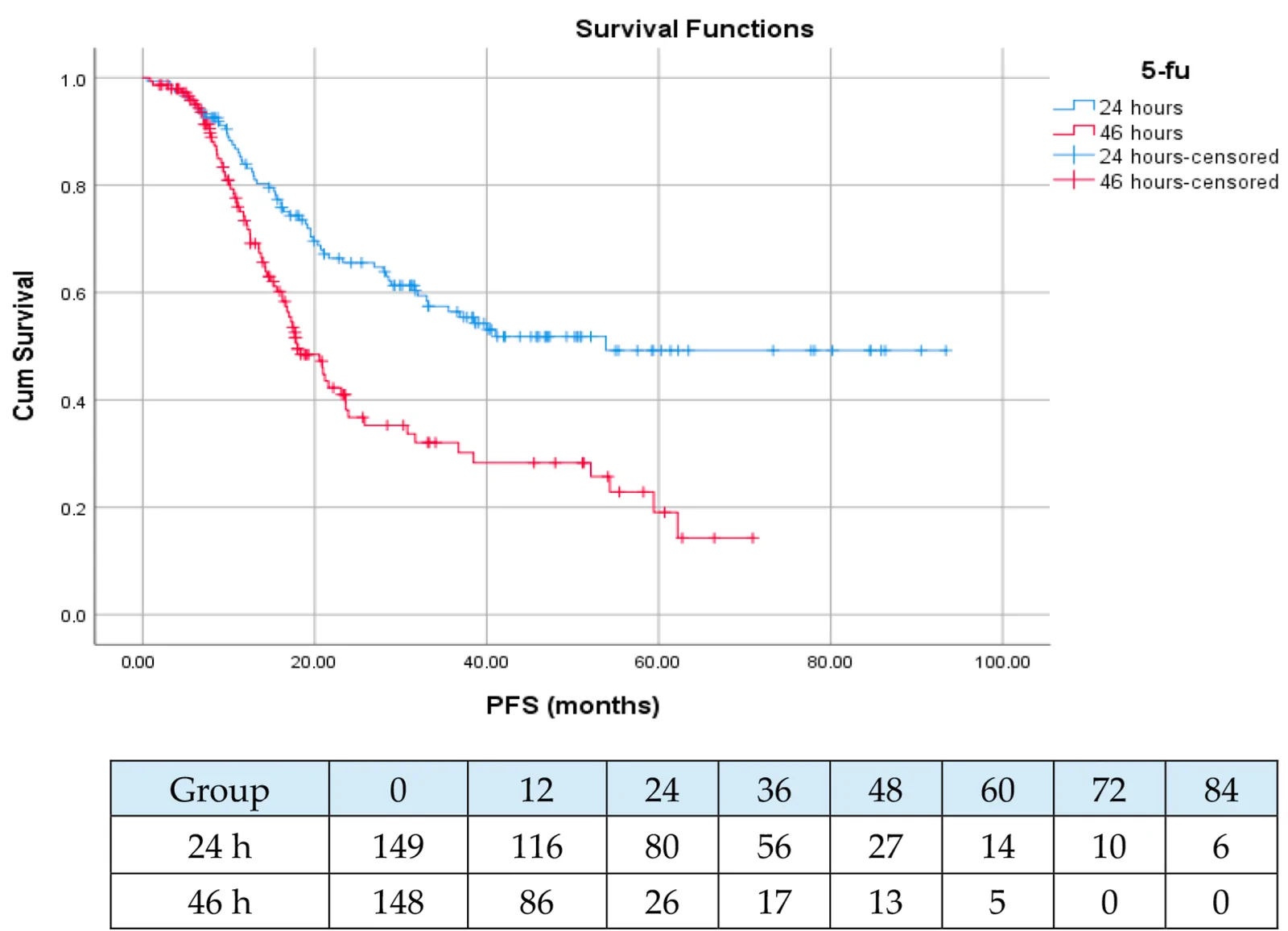
Kaplan–Meier survival curves for progression-free survival (PFS) comparing the 24 h and 46 h 5-FU infusion groups. The table beneath the curves displays the number of patients at risk at each specified time point.

According to the analysis results, the median progression-free survival (PFS) in patients receiving 24 h 5-FU was 53.87 months; however, the 95% confidence interval could not be calculated due to an insufficient number of events occurring on the survival curve. In this group, the 2-year PFS rate was 65.6%, and the 5-year PFS rate was 49.2%. In contrast, the median PFS for patients receiving 46 h 5-FU was 18.00 months (95% CI: 14.61–21.39). In this cohort, the 2-year PFS rate was 36.7%, and the 5-year PFS rate was 19.1%. A statistically significant difference in progression-free survival was observed between the groups (*p* < 0.001). Consequently, patients treated with the 24 h 5-FU infusion demonstrated significantly longer progression-free survival times and higher long-term survival rates compared to those treated with the 46 h infusion (Figure 2). The number of patients at risk at 0, 12, 24, 36, and 48 months was 149, 116, 80, 56, and 27 in the 24 h group, and 148, 86, 26, 17, and 13 in the 46 h group, respectively.

### 3.1. Pathological Response Analysis

In the logistic regression analysis performed to evaluate the factors affecting pathological response, the 46 h 5-FU infusion was found to be an independent predictive factor for the development of pathological response (OR = 2.884; 95% CI: 1.619–5.138; *p* < 0.001) (Table 2).

When the baseline tumor stage was examined, the probability of developing a pathological response was found to be significantly lower in patients with T4a–T4b disease (OR = 0.290; 95% CI: 0.115–0.731; *p* = 0.009). Similarly, as the baseline nodal stage increased, pathological response rates were found to decrease significantly. The probability of developing a pathological response was markedly lower in patients with N1 (*p* = 0.019), N2 (*p* = 0.001), and N3 (*p* = 0.005) disease (Table 2).

No significant relationship was found between pathological response and age, sex, or tumor differentiation grade.

### 3.2. Factors Affecting Overall Survival

In the Cox regression analysis, the 5-FU infusion duration was found to have no significant effect on OS (HR = 1.165; 95% CI: 0.834–1.626; *p* = 0.371).

In univariate analysis, baseline T4 stage, N3 disease, poorly differentiated tumor histology, advanced ypT stage, advanced ypN stage, positive surgical margin, and increased number of positive lymph nodes were found to be associated with poorer OS. However, in multivariate analysis, none of these variables were found to be independent prognostic factors (Table 3).

### 3.3. Factors Affecting Progression-Free Survival

In the Cox regression analysis evaluating the factors affecting PFS, 5-FU infusion duration was not found to be an independent prognostic factor in multivariate analysis (*p* = 0.105) (Table 4). However, moderately differentiated (HR = 4.005; 95% CI: 1.502–10.679; *p* = 0.006) and poorly differentiated (HR = 3.541; 95% CI: 1.328–9.439; *p* = 0.012) tumors were found to significantly increase the risk of progression. Additionally, each unit increase in the number of positive lymph nodes was found to independently increase the risk of progression (HR = 1.058; 95% CI: 1.019–1.098; *p* = 0.003). When evaluated in terms of pathological nodal stage, the risk of progression was found to be significantly increased in patients with 3–6 positive lymph nodes (HR = 2.535; 95% CI: 1.317–4.881; *p* = 0.005) (Table 4).

### 3.4. Treatment Delivery and Tolerability

Regarding neoadjuvant treatment delivery, the completion rate of the planned four cycles of preoperative FLOT was comparable between the groups, with 88% (n=132) in the 24 h group and 84.7% (n=127) in the 46 h group (p=0.412). Dose reductions of at least one chemotherapeutic agent during the preoperative phase were documented in 21.3% (n=32) of patients in the 24 h group and 28% (n=42) in the 46 h group (p=0.180). Treatment delays of more than one week occurred in 14.7% (n=22) and 18.7% (n=28) of the patients, respectively (p=0.365). Although a standardized, Common Terminology Criteria for Adverse Events (CTCAE) graded prospective toxicity registry was not consistently available across all centers due to the retrospective design, the primary clinically documented reasons for these dose modifications and treatment delays in both groups were hematological toxicities (predominantly neutropenia) and peripheral neuropathy.

## 4. Discussion

In this study, the effects of 24 h and 46 h continuous 5-FU infusion durations on pathological response and survival outcomes were evaluated in patients with gastric and gastroesophageal junction adenocarcinoma receiving perioperative FLOT therapy. The most striking finding of our study is that although pathological response rates were significantly higher in the 46 h infusion group, progression-free survival was found to be longer in the 24 h infusion group. In contrast, no significant difference was observed between the two groups in terms of overall survival.

The FLOT regimen is currently accepted as the standard perioperative treatment approach in locally advanced resectable gastric cancer [10,11]. The FLOT4-AIO study demonstrated that the FLOT regimen provided significant improvements in both pathological regression rates and overall survival [6]. Real-world data from recent years also support the efficacy of perioperative FLOT therapy [12,13].

In our study, 46 h 5-FU infusion was shown to be independently associated with pathological response. Since 5-FU is a time-dependent antimetabolite, it is thought that longer tumor exposure may increase cytotoxic efficacy. In particular, more pronounced thymidylate synthase inhibition in continuous infusion applications may theoretically provide higher tumor regression. This situation may partially explain the pathological response advantage found in favor of the 46 h infusion in our study. However, this therapeutic efficacy must be interpreted within the complex biological framework of gastric cancer differentiation. Translational evidence indicates that gastric-enriched markers like VSIG1 reflect the tumor’s differentiation state, showing strong correlation with preserved glandular architecture and epithelial stability [14]. Conversely, the loss of such differentiation markers is highly prevalent in diffuse-type, poorly differentiated, and signet ring cell carcinomas, which are characterized by heightened EMT activation and distinct lineage plasticity [5,14]. Consequently, tumors with advanced EMT phenotypes may exhibit altered pharmacodynamics and clinical heterogeneity, making them biologically less susceptible to sustained chemotherapy exposure and more prone to rapid recurrence.

However, despite the pathological response advantage, it is remarkable that progression-free survival was found to be longer in the 24 h infusion group. Under normal circumstances, the difference in infusion duration alone would not be expected to have a significant impact on survival. Therefore, it is thought that this finding may have resulted from the clinicopathological heterogeneity between the study groups.

Indeed, in our study, the 46 h infusion group notably features more advanced-stage disease, a higher number of positive lymph nodes, a higher ypN stage, and a greater proportion of signet ring cell histology. Furthermore, the rates of total gastrectomy, D2 dissection, and the removal of ≥16 lymph nodes are also significantly higher in this group. This suggests that the 46 h infusion group was initially composed of patients with biologically more aggressive tumor characteristics. Consequently, our findings suggest that the observed disparity in PFS may not stem directly from the infusion duration, but rather from the non-homogeneous baseline distribution between the study groups. This paradoxical discrepancy—where the 46 h group demonstrated superior pathological response but inferior PFS—highlights the complex interplay between treatment-induced local tumor regression and aggressive baseline tumor biology. Although prolonged 5-FU infusion (46 h) may maximize thymidylate synthase inhibition and lead to transient local tumor shrinkage, it does not overcome the adverse systemic prognosis dictated by aggressive biological features. The 46 h cohort was heavily enriched with poor prognostic factors, including poorly differentiated tumors, signet ring cell carcinoma, and advanced nodal involvement. These aggressive tumor subtypes are known to harbor high metastatic potential and propensity for early systemic recurrence, which explains why the initial local response advantage did not translate into a progression-free survival benefit. This clinical scenario represents a classic example of residual confounding, where the underlying tumor biology and disease burden ultimately override the local efficacy of the chemotherapy infusion schedule. Surgical quality and the extent of lymphadenectomy remain critical determinants of survival in gastric cancer patients undergoing perioperative FLOT. While standard D2 lymphadenectomy remains the benchmark, depending on the specific clinical scenario and nodal involvement, extended procedures such as D2+ or D3 lymphadenectomy can be scheduled. Although traditional guidelines recommend the retrieval of at least 16 lymph nodes, this concept is increasingly regarded as a minimum safety threshold rather than an optimal goal for modern oncological clearance. In this context, the adoption of minimally invasive surgery combined with advanced visualization technologies, such as indocyanine green (ICG) fluorescence imaging, has emerged as a valuable tool. The clinical application of ICG facilitates real-time lymphatic mapping, enabling safer and more precise extended lymphadenectomies while potentially reducing postoperative morbidity.

Similarly, recently published real-world data have reported that the response to FLOT therapy is closely related to tumor depth, lymph node stage, histological grade, and tumor location [12,15]. In particular, advanced T stage and poorly differentiated tumors have been shown to be associated with lower pathological response rates [15]. Consistently, in our study, baseline cT4 disease and advanced nodal stage were found to significantly decrease the probability of developing a pathological response.

The relationship between pathological response and survival is well defined in the literature. Schirren et al. demonstrated that histopathological regression achieved after neoadjuvant/perioperative treatment is one of the most important predictors of long-term survival [16]. Recently, analyses from the MATTERHORN trial also reported that achieving any degree of pathological response provides a significant advantage in terms of event-free survival [17]. Similarly, interim analyses of the KEYNOTE-585 trial demonstrated that perioperative immunotherapy combinations could increase pathological complete response rates [18]. However, the fact that the pathological response advantage in our study did not translate to PFS and OS to the same extent may be related to the prognostic imbalances between the patient groups.

In our study, multivariate analysis demonstrated that moderately and poorly differentiated tumors independently increased the risk of progression. Additionally, an increase in the number of positive lymph nodes was found to be an independent prognostic factor. These findings are consistent with the current literature [19,20]. In particular, it is well known that postoperative nodal burden is one of the most critical determinants of long-term prognosis in GC [20,21]. Similarly, recently published systematic reviews and meta-analyses have also reported that the risk of recurrence is significantly increased in patients who develop an insufficient pathological response [20].

Another notable finding is that 5-FU infusion duration could not be shown as an independent prognostic factor in terms of either OS or PFS in the multivariate analysis. This result supports the notion that infusion duration alone is not a decisive factor and that survival outcomes are primarily associated with tumor biology. Consequently, prospective randomized trials are needed to speak of a definitive superiority between 24 h and 46 h infusion applications in clinical practice.

When interpreting these findings, it is essential to distinguish between statistical significance and clinical relevance. For instance, while the difference in complete pathological response rates between the 24 h and 46 h cohorts did not reach statistical significance (p>0.05), the absolute percentage increase observed in the 46 h group may still hold clinical relevance. In neoadjuvant therapy for gastric cancer, even modest increments in tumor downstaging can facilitate R0 resections, which remains the cornerstone of curative-intent treatment. Conversely, the statistically significant superiority in PFS observed for the 24 h cohort must not be over-interpreted as a direct therapeutic benefit of the shorter infusion duration. Instead, this statistical difference is heavily confounded by the clinical reality that the 46 h group had a significantly worse baseline risk profile. Therefore, statistical models must always be contextualized with clinical judgment, as biological tumor heterogeneity ultimately dictates long-term survival outcomes regardless of statistical *p*-values.

Our study has several limitations. Primarily, its retrospective and single-center design may lead to selection bias. Additionally, there is a non-homogeneous distribution between the patient groups in terms of stage, histological subtype, and surgical characteristics. The significant baseline imbalances between the two groups—including tumor stage, nodal involvement, histological subtype, and the rates of D2 lymph node dissection—represent a major confounding factor that may have substantially influenced the observed PFS outcomes. Although we conducted multivariable logistic and Cox regression models to statistically adjust for these clinicopathological disparities, residual confounding cannot be entirely ruled out. To overcome such limitations in retrospective cohorts, future studies could employ more advanced statistical adjustment methods, such as Propensity Score Matching (PSM), to construct highly balanced treatment groups and minimize selection bias. Acknowledging this statistical imbalance is essential when interpreting our findings. Additionally, the absence of key molecular parameters, such as MSI status and HER2 expression for a subset of patients, represents another significant limitation of this study. It has been previously demonstrated that MSI status carries prognostic and predictive significance in gastric cancer [22]. However, our study is of importance as it represents one of the limited real-world datasets evaluating different 5-FU infusion durations in perioperative FLOT therapy. Furthermore, because of the retrospective multi-center design of our study, we faced difficulties in collecting standardized, comprehensive toxicity data according to CTCAE across all centers. This limited our ability to perform a detailed, survival-associated analysis of treatment modifications, delays, and specific adverse event grades. While the available data suggested similar treatment completion and dose reduction rates between the groups, the lack of granular toxicity profiles remains a limitation that should be addressed in future prospective trials.

In conclusion, our study demonstrated that 46 h 5-FU infusion was associated with a higher pathological response rate; however, this advantage did not translate into survival outcomes. It is thought that the difference observed in PFS may largely result from the clinicopathological heterogeneity between the groups. Prospective randomized trials with larger patient series are needed to determine the optimal 5-FU infusion duration in perioperative FLOT therapy.

## 5. Conclusions

In conclusion, although 46 h continuous 5-FU infusion was associated with improved pathological response rates in patients receiving perioperative FLOT therapy for gastric and gastroesophageal junction adenocarcinoma, this advantage did not translate into improved survival outcomes. The longer PFS observed in the 24 h infusion group is likely related to the baseline clinicopathological imbalances between the study groups rather than the infusion duration itself. Overall, our findings suggest that tumor biology and disease burden remain the major determinants of long-term prognosis. Prospective randomized studies with larger and more homogeneous patient populations are warranted to clarify the clinical impact of different 5-FU infusion durations within the FLOT regimen.

## Figures and Tables

**Table 1:** Demographic data.

			5-FU		*p*
		Total	24 h	46 h	
		(Mean ± SD)	(Mean ± SD)	(Mean ± SD)	
Age *		63.72 ± 10.1	63.72 ± 10.1	59.17 ± 10.12	0.001
BMI *		24.87 ± 5.19	24.87 ± 5.19	24.74 ± 4.06	0.462
		(Q25_Q75) Median	(Q25_Q75) Median	(Q25_Q75) Median	
Positive LN number **		(0_5)2	(0_4)1	(0_7)3	0.001
		n (%)	n (%)	n (%)	
Sex ***	Female	83 (27.7%)	41 (27.3%)	42 (28%)	1.000
	Male	217 (72.3%)	109 (72.7%)	108 (72%)	
Initial cT ***	T1	4 (1.5%)	4 (2.7%)	0 (0%)	0.110
	T2	48 (17.5%)	32 (21.3%)	16 (12.9%)	
	T3	160 (58.4%)	82 (54.7%)	78 (62.9%)	
	T4a	59 (21.5%)	30 (20%)	29 (23.4%)	
	T4b	3 (1.1%)	2 (1.3%)	1 (0.8%)	
Initial cN ***	N0	36 (13.1%)	14 (9.3%)	22 (17.7%)	0.067
	N1	118 (43.1%)	73 (48.7%)	45 (36.3%)	
	N2	92 (33.6%)	51 (34%)	41 (33.1%)	
	N3	28 (10.2%)	12 (8%)	16 (12.9%)	
Stage at Diagnosis ***	1	6 (2%)	2 (1.3%)	4 (2.7%)	0.046
	2A	43 (14.3%)	27 (18%)	16 (10.7%)	
	2B	46 (15.3%)	28 (18.7%)	18 (12%)	
	3	199 (66.3%)	92 (61.3%)	107 (71.3%)	
	4A	6 (2%)	1 (0.7%)	5 (3.3%)	
Tumor Localization ***	GEJ	35 (12.8%)	11 (7.3%)	24 (19.4%)	0.028
	Cardia	59 (21.5%)	36 (24%)	23 (18.5%)	
	Fundus	24 (8.8%)	16 (10.7%)	8 (6.5%)	
	Corpus	57 (20.8%)	29 (19.3%)	28 (22.6%)	
	Antrum	82 (29.9%)	45 (30%)	37 (29.8%)	
	Pylorus	17 (6.2%)	13 (8.7%)	4 (3.2%)	
Pathology ***	Adenocarcinoma	247 (82.3%)	130 (86.7%)	117 (78%)	0.007
	Signet ring cell carcinoma	53 (17.7%)	20 (13.3%)	33 (22%)	
Lauren ***	Intestinal	144 (52.6%)	104 (69.3%)	40 (32.3%)	0.001
	Diffuse	50 (18.2%)	38 (25.3%)	12 (9.7%)	
	Unknown	80 (29.2%)	8 (5.3%)	72 (58.1%)	
Grade ***	Well-differentiated	42 (15.4%)	24 (16%)	18 (14.8%)	0.008
	Moderately differentiated	111 (40.8%)	68 (45.3%)	43 (35.2%)	
	Poorly differentiated	119 (43.8%)	58 (38.7%)	61 (50%)	
Surgery ***	Subtotal gastrectomy	101 (36.9%)	72 (48%)	29 (23.4%)	0.001
	Total gastrectomy	173 (63.2%)	78 (52%)	95 (76.6%)	
ypT ***	0	36 (12%)	17 (11.3%)	19 (12.7%)	0.093
	1	38 (12.7%)	26 (17.3%)	12 (8%)	
	2	35 (11.7%)	16 (10.7%)	19 (12.7%)	
	3	116 (38.7%)	55 (36.7%)	61 (40.7%)	
	4a	71 (23.7%)	36 (24%)	35 (23.3%)	
	4b	4 (1.3%)	0 (0%)	4 (2.7%)	
LN Dissection ***	D2	171 (62.4%)	64 (42.7%)	107 (86.3%)	0.001
	D1	103 (37.6%)	86 (57.3%)	17 (13.7%)	
Number of Harvested LNs ***	≥16	178 (59.3%)	57 (38%)	121 (80.7%)	0.001
	<16	122 (40.7%)	93 (62%)	29 (19.3%)	
ypN ***	0	108 (36%)	68 (45.3%)	40 (26.7%)	0.001
	1–2 lymph nodes	57 (19%)	25 (16.7%)	32 (21.3%)	
	3–6 lymph nodes	75 (25%)	40 (26.7%)	35 (23.3%)	
	7 and more lymph nodes	60 (20%)	17 (11.3%)	43 (28.7%)	
ypTNM ***	0	26 (8.7%)	13 (8.7%)	13 (8.7%)	0.181
	1	49 (16.3%)	31 (20.7%)	18 (12%)	
	2	85 (28.3%)	38 (25.3%)	47 (31.3%)	
	3	140 (46.7%)	68 (45.3%)	72 (48%)	
Surgical Margins ***	Negative	285 (95%)	143 (95.3%)	142 (94.7%)	0.639
	Positive	15 (5%)	7 (4.7%)	8 (5.3%)	
HER2 IHC ***	Negative	252 (84%)	129 (86%)	123 (82%)	0.008
	Positive	32 (10.7%)	19 (12.7%)	13 (8.7%)	
	Unknown	16 (5.3%)	2 (1.3%)	14 (9.3%)	
MSI status ***	Stable	196 (65.3%)	87 (58%)	109 (72.7%)	0.001
	High	7 (2.3%)	1 (0.7%)	6 (4%)	
	Unknown	97 (32.3%)	62 (41.3%)	35 (23.3%)	

* = *t* test, ** = Mann–Whitney, *** = Ki-square test; 5-FU: 5-fluorouracil; SD: standard deviation; Q: Quartile; BMI: Body Mass Index; LN: lymph node; cT: clinical tumor stage; cN: clinical node stage; GEJ: gastroesophageal junction; ypT: post-neoadjuvant therapy pathological tumor stage; ypN: post-neoadjuvant therapy pathological node stage; ypTNM: post-neoadjuvant therapy pathological tumor, node and metastasis stage; HER2: human epidermal growth factor receptor 2; IHC: immunohistochemistry; MSI: Microsatellite Instability.

**Table 2:** Investigation of the effects of variables on pathological response.

	Univariate			Multivariate		
	OR	95% CI	*p*	OR	95% CI	*p*
5-FU (46 h)	1.981	1.245_3.151	**0.004**	2.884	1.619_5.138	**0.001**
Age	0.993	0.971_1.016	0.550			
Sex (Male)	0.844	0.503_1.418	0.522			
Baseline cT (T3)	0.562	0.275_1.148	0.114	0.470	0.207_1.065	0.071
Baseline cT (T4a_T4b)	0.246	0.11_0.548	**0.001**	0.290	0.115_0.731	0.009
Baseline cN (N1)	0.064	0.008_0.481	0.008	0.086	0.011_0.664	0.019
Baseline cN (N2)	0.027	0.004_0.202	**0.000**	0.034	0.004_0.268	**0.001**
Baseline cN (N3)	0.026	0.003_0.214	**0.001**	0.045	0.005_0.393	**0.005**
Grade (Moderately differentiated)	0.471	0.198_1.118	0.088	0.671	0.253_1.779	0.422
Grade (Poorly differentiated)	0.297	0.128_0.688	**0.005**	0.403	0.153_1.064	0.067

Logistic regression analysis; 5-FU: 5-fluorouracil; OR: Odds Ratio; CI: Confidence Interval; cT: clinical tumor stage; cN: clinical node stage.

**Table 3:** Investigation of the effects of variables on OS.

	Univariate			Multivariate		
	HR	95% CI	*p*	HR	95% CI	*p*
5-FU (46 h)	1.165	0.834_1.626	0.371			
Age	0.988	0.972_1.005	0.159			
Sex (Male)	0.911	0.634_1.31	0.616			
Baseline cT (T3)	1.677	0.974_2.886	0.062	1.008	0.546_1.86	0.980
Baseline cT (T4a_T4b)	2.851	1.603_5.071	**0.000**	1.367	0.704_2.655	0.356
Baseline cN (N1)	1.407	0.752_2.633	0.285	0.768	0.376_1.569	0.469
Baseline cN (N2)	1.555	0.815_2.966	0.180	0.570	0.262_1.238	0.156
Baseline cN (N3)	2.723	1.326_5.591	0.006	0.708	0.293_1.713	0.444
Grade (Moderately differentiated)	1.769	0.908_3.446	0.094	1.437	0.69_2.996	0.333
Grade (Poorly differentiated)	2.692	1.426_5.08	**0.002**	1.753	0.864_3.556	0.120
ypT (1)	1.989	0.7_5.651	0.197	2.128	0.724_6.258	0.170
ypt (2)	1.313	0.429_4.021	0.633	1.069	0.335_3.415	0.910
ypT (3)	3.343	1.344_8.316	0.009	2.115	0.786_5.69	0.138
ypT (4a)	5.217	2.082_13.07	**0.000**	2.454	0.886_6.799	0.084
ypN (1–2 lymph node)	1.906	1.128_3.222	0.016	1.647	0.844_3.212	0.144
ypN (3–6 lymph node)	2.264	1.417_3.617	**0.001**	1.786	0.939_3.397	0.077
ypN (7 and more lymph nodes)	3.800	2.368_6.096	**0.000**	2.154	0.943_4.918	0.069
Surgical Margins	1.835	1.013_3.324	0.045	1.381	0.709_2.688	0.343
Positive LN number	1.053	1.034_1.073	**0.000**	1.019	0.98_1.06	0.344

Cox regression test; HR: Hazard Ratio; CI: Confidence Interval; 5-FU: 5-fluorouracil; cT: clinical tumor stage; cN: clinical node stage; ypT: Post-neoadjuvant therapy pathological tumor stage; ypN: Post-neoadjuvant therapy pathological node stage; LN: Lymph node.

**Table 4:** Investigation of the effects of variables on PFS.

	Univariate			Multivariate		
	HR	95% CI	*p*	HR	95% CI	*p*
5-FU (46 H)	1.950	1.399_2.718	**0.000**	1.383	0.934_2.047	0.105
Age	0.976	0.96_0.991	**0.002**	0.988	0.971_1.005	0.153
Sex (Male)	0.952	0.669_1.354	0.783			
Baseline cT (T3)	2.442	1.383_4.311	**0.002**	1.358	0.737_2.503	0.327
Baseline cT (T4a-T4b)	3.850	2.098_7.065	**0.000**	1.628	0.814_3.253	0.168
Baseline cN (N1)	3.236	1.398_7.491	0.006	1.582	0.641_3.903	0.320
Baseline cN (N2)	3.787	1.617_8.867	**0.002**	1.222	0.472_3.164	0.680
Baseline cN (N3)	7.678	3.056_19.293	**0.000**	1.177	0.403_3.437	0.765
Grade (Moderately differentiated)	4.846	1.929_12.174	**0.001**	4.005	1.502_10.679	0.006
Grade (Poorly differentiated)	7.098	2.872_17.545	**0.000**	3.541	1.328_9.439	0.012
ypT (1)	1.208	0.46_3.174	0.702	1.479	0.53_4.126	0.455
ypT (2)	1.713	0.683_4.297	0.251	1.296	0.486_3.461	0.604
ypT (3)	2.941	1.347_6.423	0.007	1.244	0.515_3.003	0.627
ypT (4a)	5.145	2.341_11.31	**0.000**	1.810	0.722_4.535	0.206
Positive LN number	1.071	1.054_1.087	**0.000**	1.058	1.019_1.098	**0.003**
ypN (1–2 lymph nodes)	2.444	1.407_4.245	**0.002**	1.924	0.961_3.853	0.065
ypN (3–6 lymph nodes)	3.808	2.337_6.205	**0.000**	2.535	1.317_4.881	**0.005**
ypN (7 or more lymph nodes)	7.201	4.388_11.815	**0.000**	2.277	0.99_5.236	0.053
Surgical Margins	1.607	0.868_2.974	0.131			

Cox regression test; HR: Hazard Ratio; CI: Confidence Interval; 5-FU: 5-fluorouracil; cT: clinical tumor stage; cN: clinical node stage; ypT: Post-neoadjuvant therapy pathological tumor stage; LN: Lymph node; ypN: Post-neoadjuvant therapy pathological node stage.

## Data Availability

The dataset linked to this research cannot be shared publicly, strictly adhering to national patient privacy and confidentiality rules. Nonetheless, anonymized data can be supplied if a suitable request is made and subsequently approved by the authors and the ethics committee.

## Abbreviations

The following abbreviations are used in this manuscript:

5-FU	5-Fluorouracil
ECF	Epirubicin, Cisplatin, 5-FU
PFS	Progression-Free Survival
OS	Overall Survival
FLOT	Fluorouracil, Leucovorin, Oxaliplatin and Docetaxel
T	Tumor
N	Node
BMI	Body Mass Index
HER 2	Human Epidermal Growth Factor Receptor 2
MSI	Microsatellite Instability
AJCC	American Joint Committee on Cancer
TNM	Tumor Node Metastasis
SD	Standard Deviation
LN	Lymph Node
cT	Clinical Tumor Stage
cN	Clinical Node Stage
GC	Gastric Cancer
GEJ	Gastroesophageal Junction
ypT	Post-neoadjuvant Therapy Pathological Tumor Stage
ypN	Post-neoadjuvant Therapy Pathological Node Stage
ypTNM	Post-neoadjuvant Therapy Pathological Tumor, Node and Metastasis Stage
IHC	Immunohistochemistry
OR	Odds Ratio
CI	Confidence Interval
HR	Hazard Ratio
EMT	Epithelial–mesenchymal transition
VSIG1	V-set and immunoglobulin domain-containing 1
CTCAE	Common Terminology Criteria for Adverse Events
ICG	Indocyanine green
PSM	Propensity Score Matching
